# Consumption of Selected Healthy and Unhealthy Food Groups and Associations With Nutritional Status Among Children 2–5 Years of Age in Northern Ghana

**DOI:** 10.1111/mcn.70126

**Published:** 2025-11-18

**Authors:** Emily R. Becher, Sika M. Kumordzie, Jennie N. Davis, Charles D. Arnold, K. Ryan Wessells, Xiuping Tan, Ahmed D. Fuseini, Katherine P. Adams, Marjorie J. Haskell, Stephen A. Vosti, Seth Adu‐Afarwuah, Reina Engle‐Stone

**Affiliations:** ^1^ Institute for Global Nutrition University of California, Davis Davis California USA; ^2^ Department of Nutrition University of California, Davis Davis California USA; ^3^ Department of Nutrition and Food Science University of Ghana Legon Accra Ghana; ^4^ Department of Agricultural and Resource Economics University of California Davis California USA

**Keywords:** child, dietary patterns, Ghana, malnutrition, nutritional status, preschool, snacks, sugar‐sweetened beverages

## Abstract

Poor diet quality may contribute to the multiple forms of malnutrition among children in Ghana. This cross‐sectional study (1) described the prevalence and frequency of consumption of fruits, vegetables, sugar‐sweetened beverages (SSB), salty snacks and sweet snacks among children 2–5 years in northern Ghana; (2) identified factors associated with consumption; and (3) examined relationships between consumption and nutritional status. Households were recruited from urban and rural clusters in Tolon and Kumbungu districts. Children's (2–5 years; *n* = 243) dietary data were collected using a questionnaire modelled after the WHO STEPS tool. We assessed children's height, weight, haemoglobin and micronutrient biomarker (*n* = 125) concentrations. We used multi‐variable logistic and linear regression to identify individual, maternal and household factors predicting consumption of the food groups and relationships between consumption and nutritional status. In a typical week, most children consumed vegetables (98%), sweet snacks (81%) and fruits (76%); 50% consumed salty snacks and 46% consumed SSB. Average number of servings consumed weekly, mean (SD), was: 7.9 (7.3) vegetables, 2.9 (2.5) sweet snacks, 2.6 (3.9) SSB, 1.8 (1.7) fruits and 1.2 (1.7) salty snacks. Children in households with higher food insecurity were more likely to report consumption of all food groups (> 0 servings fruits, SSB, salty snacks and sweet snacks; ≥ 7 servings vegetables weekly), but other characteristics did not consistently predict consumption. Few associations were found between consumption and nutritional status. Interventions to increase fruit and vegetable intake to align with guidelines, while avoiding SSB and snack foods, are needed.

## Summary

- Diets of children 2–5 years in Kumbungu and Tolon districts, northern Ghana, were severely inadequate with respect to recommended consumption of fruits and vegetables, with only ~11% meeting WHO recommendations.
- Half of children reported consumption of sugar‐sweetened beverages in a typical week, and 87% consumed sweet or salty snacks, but average servings consumed in a typical week were < 3 for each food group.
- These findings suggest the need to increase fruit and vegetable consumption among young children without increasing sugar‐sweetened beverage or sweet and salty snack consumption in these communities.

## 1 Introduction

Many children in low‐ and middle‐income countries (LMICs) face the coexistence of multiple forms of malnutrition (United Nations Children's Fund et al. 2023). Children under 5 residing in LMICs account for 76% of child wasting, 64% of stunting and 42% of overweight and obesity, globally (Development Initiatives 2022); an estimated 56% of pre‐school age children (6–59 months) globally and 62% in Sub‐Saharan Africa have at least one micronutrient deficiency (vitamin A, iron or zinc) (Stevens et al. 2022).

Diet quality and changing dietary patterns have been implicated in the multiple forms of malnutrition observed to co‐occur in LMICs (Popkin et al. 2020; Popkin and Ng 2022; Wells et al. 2020). A diverse and nutrient‐dense diet is important for the growth and development of children under 5. The World Health Organization guidelines on healthy diets and the 2009 Ghana Dietary Guidelines and United States Dietary Guidelines recommend an energy‐balanced diet, with a variety of fruits, vegetables, legumes, nuts and whole grains for children under 5 to meet their nutrient needs and promote growth and development (Ministry of Food and Agriculture 2023; Ministry of Health 2010; WHO, 2015; World Health Organization 2023). Yet, the diets of young children in LMICs are often predominantly reliant on starchy staples and low in nutrient‐dense foods such as fruits, vegetables and animal‐sourced foods; such diets often fail to meet the high nutritional needs of young children (Black et al. 2013; Dewey 2013; Kupka et al. 2020; Osendarp et al. 2016). Fruits and vegetables are an important component of the diet as they provide micronutrients and dietary fibre, and their consumption decreases risk of non‐communicable diseases (World Health Organization 2011; Yip et al. 2019). The WHO recommends that children aged 2–5 consume 250 g per day of fruits and vegetables (World Health Organization 2023). However, a recent analysis of Demographic and Health Surveys found that in West and Central Africa, 56.1% of children 6–23 months consumed zero fruits or vegetables on the previous day (C. K. Allen et al. 2023), suggesting that many children may not meet recommendations.

A nutrition transition, characterised by increased availability and consumption of sugar‐sweetened beverages (SSB) and processed snack foods, has also been identified as a potential contributor to multiple forms of malnutrition (Bosu 2015; Monteiro et al. 2013; Popkin et al. 2020; Popkin and Ng 2022). Specifically, increased consumption of SSB and snack foods may contribute to the increasing prevalence of overweight and obesity and related non‐communicable diseases like Type 2 diabetes and hypertension observed in LMIC (Popkin et al. 2012; Popkin and Gordon‐Larsen 2004; Popkin and Ng 2022). It has been hypothesised that consumption of SSB and ultra‐processed snack foods may displace consumption of nutrient‐dense options, which is of concern among young children who may have difficulty reaching their needs for some micronutrients due to their high nutrient requirements (Pries et al. 2019). While there are no specific intake guidelines, the WHO and Ghana Dietary Guidelines recommend limiting consumption of SSB and ‘unhealthy’ snack foods (high in sugar, salt and saturated fat) (Ministry of Food and Agriculture 2023; Ministry of Health 2010; WHO, 2015).

Ghana is undergoing such a nutrition transition, and multiple forms of malnutrition coexist in the country (Bosu 2015). Nationally, it is estimated that 7% of children under 5 are wasted, 18% are stunted, and 21.5% of children 6–59 months have iron deficiency (University of Ghana et al. 2017). However, 19% of adult women experience obesity (Development Initiatives 2022), and over the past 20 years, there has been an increase in availability and consumption of processed foods in urban centres like Accra (Holdsworth et al. 2020; Kushitor 2023; Rousham et al. 2022). The Northern Region of Ghana reports the highest prevalence of stunting, anaemia and vitamin A deficiency in the country, and also suffers from high rates of poverty and food insecurity (Ghana Statistical Survey & UNICEF 2019). While undernutrition among children is well documented in the Northern Region, extent of the nutrition transition is uncertain (Ghana Statistical Survey & UNICEF 2019). Limited information is available on consumption of fruits, vegetables and processed foods, like SSB and snack foods among pre‐school age children.

Therefore, in this study we used cross‐sectional data from a pilot survey conducted in the Tolon and Kumbungu districts in the Northern Region of Ghana to assess consumption of fruits, vegetables, SSB and salty and sweet snacks among pre‐school age children. The objectives of this study were: (1) to describe the prevalence and frequency of consumption of fruits, vegetables, SSB and salty and sweet snacks, i.e., food groups) among children 2–5 years of age; (2) identify individual, maternal and household predictors of the consumption of these food groups; and (3) determine whether consumption of these food groups predicts nutritional status (i.e., anthropometric measures and micronutrient status).

## 2 Methods

### 2.1 Overview

This analysis used data that were collected as part of a cross‐sectional pilot survey (Adu‐Afarwuah et al. 2025) conducted for the Condiment Micronutrient Innovation Trial (CoMIT) project, NCT04632771 (Engle‐Stone et al. 2024). The primary aim of the pilot survey was to assess the micronutrient status of women of reproductive age (WRA, non‐pregnant, non‐lactating women [LW], 15–49 years) and children 2–5 years of age, as well as the micronutrient content of breast milk among LW (non‐pregnant LW 4–18 months postpartum and 15–49 years). The study took place between November 2020 and December 2020, at the end of the rainy season and the beginning of the dry season in northern Ghana. Results for the primary aims of the pilot are presented elsewhere (Adu‐Afarwuah et al. 2025). The present analysis used data on dietary intake, nutritional status and household characteristics among children enroled in the pilot survey. The study protocol was approved by the Ghana Ethical Review Committee and the University of California, Davis, Institutional Review Board.

### 2.2 Recruitment

The study aimed to recruit a total of 250 WRA, 250 LW and 250 children from the Tolon and Kumbungu districts of northern Ghana. Participants were recruited from randomly selected urban (*n* = 7 per district) and rural/semi‐urban clusters (*n* = 7 per district), as defined by Ghana Statistical Service. Within selected clusters, a random walk method was used to select households for recruitment. Multiple physiological groups could be enroled from one household but only one participant per physiological group could be enroled (e.g., a household could have one child, one WRA and one LW enroled but not two children). Children were enroled if they were 2–5 years of age and had a signed informed consent form by a parent or guardian. The written informed consent process was completed with one parent/guardian by a trained enumerator. The consent form was written in English but presented in Dagbani in the presence of a neutral witness fluent in English and Dagbani; the parent/guardian could sign or thumbprint the consent form. Children were excluded if any of the following exclusion criteria applied: Severe illness warranting hospital referral, chronic severe medical condition (e.g., malignancy) or congenital anomalies (e.g., spina bifida) requiring frequent medical attention or potentially interfering with nutritional status, parent or primary caregiver unable to give informed consent due to impaired decision‐making abilities, or current participation in a clinical trial.

### 2.3 Data Collection

At the recruitment visit, the head of household, participants (WRA, LW), or parents or caregivers (hereafter, caregivers) of participating children (if not the WRA or LW) responded to questionnaires that gathered data on household and individual demographic characteristics. Caregivers of participating children also responded to individual‐level questionnaires on the child's health history and diet. Household food insecurity was measured with the USAID Household Food Insecurity and Access Scale questionnaire (Jennifer Coates, Anne Swindale 2016). Household assets, animal ownership and demographic characteristics were measured with a questionnaire adapted from the 2014 Demographic Health Survey and Fortification Assessment Coverage Toolkit: Household Assessment Template with locally relevant options (Friesen et al. 2019; Ghana Statistical Service [GSS] 2015). All questionnaires were piloted and adapted to the local context with feedback from the local staff incorporated; this process included translating all questionnaires into the local language, Dagbani, back‐translating into English to confirm the translation, and testing in non‐participating communities.

### 2.4 Dietary Data

Dietary data were collected using adapted dietary questions from the WHO STEPwise approach to non‐communicable disease risk factor surveillance (STEPS) (World Health Organization 2017). The STEPS questionnaire was designed to collect and monitor data on key non‐communicable disease risk factors among adults 18–69 years of age, including dietary risk factors (specifically consumption of fruits, vegetables, SSB and salty and sweet snacks), and has been used in more than 100 countries (Riley et al. 2016; World Health Organization 2017). This simplified diet questionnaire was selected to capture information on intake of food groups relevant to the nutrition transition and non‐communicable disease risk while minimising participant burden. Additional information is now available on validity of tools to assess minimum dietary diversity and diet quality of children 2–5 years of age (Diop et al. 2021), but, at the time of the study, the 2021 publication on infant and young child feeding indicators (IYCF) (World Health Organization & United Nations Children's Fund [UNICEF] 2021) and the Diet Quality Questionnaire (Global Diet Quality Project 2021) were not available. We chose to adapt the STEPS diet questions rather than the prior version of the IYCF because the STEPS questionnaire included information on SSB and unhealthy snacks. For this study, the STEPS diet questions were adapted to include examples of local foods for each group (e.g., papaya, cooked local leafy green vegetables, biscuits, toffee, sugar‐sweetened fruit‐flavoured juice (kalyppo), File S1).

In response to the modified dietary questions from the STEPS questionnaire, caregivers reported the number of days in a typical week that the participating child consumed fruit, vegetables, SSB and salty and sweet snacks and, if the food group was consumed, the number of servings consumed on a typical day. Serving sizes of fruits and vegetables were based on the WHO STEPS manual; serving sizes for SSB and snack foods were based on the FDA Reference Amounts Customarily Consumed (United States Food and Drug Administration 2018) for children, as the WHO STEPS manual did not provide a standard serving size. A serving of fruits and vegetables was defined as 80 g, a serving of SSB (hot or cold) was defined as 120 mL and a serving of salty and sweet snacks was 20 g. Showcards with photos of standard serving sizes of common locally consumed vegetables, fruits, SSBs and salty and sweet snacks, including examples like biscuits and fruit‐flavoured drinks that are commonly consumed by children, were created to assist participants in estimating the number of servings consumed (File S2).

### 2.5 Nutritional Status Data

Anthropometric data and venous blood samples were collected at mobile data collection sites. Height and weight were measured by trained anthropometrists. Standing height (SECA 217) was measured with 0.1 cm precision, and weight (SECA 874) was measured to 50 g precision. For the weight measurement, caregivers were asked to remove all the child's clothing except for a diaper. All measures were collected in duplicate and the average of the two measurements was calculated per participant for each outcome. If the two measurements differed by more than 0.1 kg (weight), 0.5 cm (height), the measurement was repeated, and the two closest measurements were retained for analysis. Weight‐for‐height *z*‐score (WHZ) and height‐for‐age *z*‐score (HAZ) were calculated according to WHO growth standards (World Health Organization 2006). Venous blood samples (up to 6 mL into trace‐element‐free BD vacutainer plastic serum tubes) were collected from children for immediate analysis of haemoglobin and malaria, and aliquots of serum were retained for analysis of micronutrient biomarkers. Haemoglobin was measured using Hemocue 301 (HemoCue AB, Angelholm, Sweden) and malaria was measured with a Malaria Rapid Diagnostic Test (SD Bioline Malaria Ag. P. falciparum/Pan, Abbott Diagnostrics, Chicago, IL). Micronutrient biomarkers measured were serum retinol (Bieri et al. 1979), serum B‐12 (Engle‐Stone et al. 2017), serum ferritin, serum transferrin receptor, retinol binding protein (Erhardt et al. 2004) and serum zinc (Killilea and Ames 2008). For this analysis, one biomarker was used for each nutrient: serum retinol, serum ferritin, serum B12 and serum zinc. Markers of inflammation measured were C‐reactive protein (CRP) (Erhardt et al. 2004) and alpha‐1‐acid glycoprotein (AGP) (Erhardt et al. 2004). If a venous sample could not be obtained, a capillary sample was used for haemoglobin and malaria assessment (29% of children), and micronutrient biomarkers were not assessed. Serum ferritin, serum zinc and serum retinol values were adjusted for inflammation (CRP and/or AGP) following the BRINDA correction method (Luo et al. 2023). Additional details of micronutrient analysis are reported elsewhere (Adu‐Afarwuah et al. 2025).

### 2.6 Micronutrient Deficiency Index

To assess micronutrient status using multiple markers, a micronutrient deficiency index was constructed by summing the number of micronutrients for which a child met criteria for deficiency (low inflammation adjusted serum retinol (< 0.70 µmol/L) (World Health Organization 2011); low serum B‐12 (< 221 pmol/L) (L. H. Allen et al. 2018); low inflammation‐adjusted serum ferritin (< 12 µg/L) (World Health Organization 2020a); low inflammation‐adjusted serum zinc (< 65 µg/dL) (Brown et al. 2004) and dividing it by the total number of micronutrient biomarkers assessed. For example, if a child had three indicators measured and two were low, their micronutrient deficiency index would be 2/3 or 0.66. Children with three or four biomarkers measured were included in the micronutrient deficiency index calculation, and children were excluded from this index if they had two or fewer micronutrient indicators measured (38% of children). A categorical version of the index was created using a cut point that identified the lowest quintile of the index, equivalent to a micronutrient deficiency index of ≥ 0.75.

### 2.7 Analysis

A statistical analysis plan was posted before data analysis (https://osf.io/t3zrn/). Data analysis was completed with SAS 9.4 (Cary, NC) and STATA SE 16 (StataCorp LLC, College Station, TX).

We created a binary consumption variable defined as consuming > 0 servings in a typical week of each food group. For vegetables, because consumption was common with the average consumption reported as ~7 servings per week, we also created a binary consumption variable defined as 7 or more servings in a typical week. The number of servings consumed in a typical week for each food group was calculated as the number of days in a typical week a food was consumed multiplied by the number of servings consumed on one of those days. To approximate the number of servings consumed per day, the estimated number of servings per week was divided by 7.

For the first objective of the study, the prevalence of any consumption of each food group in a typical week was estimated with 95% confidence intervals. For vegetables, we also estimated the prevalence of consumption ≥ 7 servings/week. The frequency of servings consumed in a typical week of each food group was estimated as mean (SD) and 95% confidence intervals. The WHO recommends children consume > 250 g per day of fruits and vegetables (World Health Organization 2023), which is approximately 3 servings. The percent of children not reaching WHO‐recommended intake of fruits and vegetables per day was estimated with 95% confidence intervals. Estimated consumption of SSB and snack foods relative to recommendations was not estimated as there are no specific quantitative intake guidelines for these food groups (World Health Organization 2020b, 2023).

To identify individual, maternal and household characteristics associated with consumption of each food group we used logistic regression in two stages. Outcome variables for the regression models were the binary consumption variables (any/no consumption for fruits, SSB, salty and sweet snacks and ≥ 7 servings/week for vegetables); the predictor variables were individual (e.g., child age and sex), maternal (e.g., maternal age and education) and household (e.g., food insecurity, asset index, household size) characteristics. Potential predictors were selected using a conceptual model of the hypothesised relationships based on theoretical relationships between individual, maternal and household factors contributing to malnutrition (UNICEF 2021) (Figure S1). All models included the predictor of interest and controlled for district (Kumbungu/Tolon) and residence area (urban/rural), and cluster as a random effect. First, minimally adjusted models separately estimated the association between the outcome and each potential predictor (e.g., fruit consumption and child age). Then, predictors associated with the outcome (*p* < 0.1) were included in multivariable models to assess the simultaneous associations between predictors and the outcome. All predictors associated (*p* < 0.1) with any one of the food categories were included in all multivariable models. Child sex and child age were controlled for in all multivariable models, regardless of association in the minimally adjusted model, as they are often found to be related to dietary intake and nutritional status (Abebe et al. 2021; Agyemang et al. 2023; Gonete et al. 2024; Kang et al. 2023).

To determine whether individual components of the diet (consumption variables described above) predicted nutritional status we used nutritional status indicators WHZ, wasting (defined as WHZ < −2 SD) (World Health Organization 2006), HAZ, stunting (defined as HAZ < −2 SD) (World Health Organization 2006), haemoglobin concentration (g/L), anaemia (hb < 110 g/L) (World Health Organization 2024), micronutrient deficiency index and categorical micronutrient deficiency index. Additional covariates included in the multivariable models were selected using a conceptual model of the hypothesised relationships between consumption and nutritional status, accounting for household and individual factors (Figure S1). Associations with continuous outcomes (WHZ, HAZ, haemoglobin concentration, micronutrient deficiency index) were assessed with linear regression; binary outcomes (wasting, stunting, anaemia status, categorical micronutrient deficiency index) were assessed using logistic regression. All models included the predictor of interest, controls for district (Kumbungu/Tolon) and residence area (urban/rural), as well as a random effect of cluster. First, minimally adjusted models estimated the observed association between the predictor and the outcome. Predictors associated with the outcome (*p* < 0.1) were included in the fully adjusted multivariable model to assess the simultaneous association with the outcome. If a potential covariate was associated with either the categorical or continuous outcome the covariate was included in the fully adjusted multivariable models for both versions of that outcome.

### 2.8 Ethical Statement

The study was approved by the Ghana Ethical Review Committee and the UC Davis Institutional Review Board (#1536100).

## 3 Results

### 3.1 Household, Caregiver and Child Characteristics

A total of 248 children consented, 243 children completed individual questionnaires and 238 children completed anthropometric measurements and biospecimen collection (Figure S2). The average household size was 13 people, and 77% of households reported moderate/severe food insecurity in the past month. (Table 1). Approximately 56% of households had access to an improved water source (defined according to Joint Monitoring Programme as piped water and non‐piped protected supplies) (World Health Organization/UNICEF 2022) and 31% reported access to improved sanitation facilities (defined according to Joint Monitoring Programme as networked and on‐site sanitation that separate excreta from human contact) (World Health Organization/UNICEF 2022). Maternal education was low among the sample, with nearly 80% of women reporting completing no formal education.

**Table 1:** Summary of characteristics of households, mothers and children 2–5 years of age who participated in the CoMIT pilot survey in the Tolon and Kumbungu districts, northern Ghana.

	*N*	*n* (%) or mean (SD)
Characteristics of households and mothers		
Household location		
Rural	243	122 (50.2%)
Urban		121 (49.8%)
Household district		
Kumbungu	243	120 (49.4%)
Tolon		123 (50.6%)
Primary household religion		
Islam	243	242 (99.6%)
Household ethnic group		
Mole‐Dagbani	243	241 (99.2%)
Highest level of education completed by any household member		
None	243	66 (27.2%)
Pre‐school		3 (1.2%)
Primary		32 (13.2%)
Secondary		103 (42.4%)
Higher than secondary		39 (16.1%)
Household size	243	12.5 (6.1)
Number of children < 5 years	243	1.97 (1.17)
Household food insecurity score^a^	243	6.09 (3.84)
Household food insecurity^a^		
None	243	13 (5.4%)
Mild		42 (17.3%)
Moderate		131 (53.9%)
Severe		57 (23.5%)
Access to improved water source^a^	243	136 (56.0%)
Improved sanitation^a^	243	75 (30.9%)
Maternal age (years)	241	33 (9.3)
Highest level of education completed by the mother		
None	241	195 (79.6%)
Pre‐school		4 (1.6%)
Primary		14 (5.7%)
Secondary		29 (11.8%)
Higher than secondary		3 (1.2%)
Characteristics of children		
Child age (months)	243	41.2 (11.3)
Child sex		
Male	238	122 (51.3%)
Currently breastfed	243	10 (4.1%)
Reported fever in previous 7 days	236	15 (6.4%)
Reported diarrhoea in previous 7 days	236	76 (32.2%)
Recent morbidity (fever and/or diarrhoea in previous 7 days)	236	81 (34.3%)
Received vitamin A capsule in past 6 months	202	50 (24.5%)
Consumed MNP in past 30 days	240	3 (1.3%)
Consumed any other vitamin or mineral supplement in past 30 days (non‐MNP)	240	20 (8.3%)
Height for age *z*‐score^b^	233	−1.3 (1.5)
Stunted^b^	233	73 (31.3%)
Weight for height *z*‐score^b^	233	−0.53 (0.92)
Wasted^b^	233	11 (4.7%)
Haemoglobin (g/L)	232	111.4 (15.4)
Anaemia^b^	232	86 (37.1%)
Low serum retinol^b^	145	41 (28%)
Low serum B12^b^	139	27 (19%)
Low serum ferritin^b^	163	94 (58%)
Low serum zinc^b^	164	111 (68%)
Micronutrient Deficiency Index^b^	154	44.2 (23.9)

Abbreviations: CoMIT, Condiment Micronutrient Innovation Trial; MNP, micronutrient powder; g/dL, grams/decilitre.

^a^ Food insecurity was measured with the USAID Household Food Insecurity and Access Scale questionnaire (USAID HFIAS). Improved water source and improved toilet were defined according to the WHO/UNICEF Joint Monitoring Programme (WHO/UNICEF Joint Monitoring Water/Sanitation).

^b^ Height for age *z*‐score (HAZ) and weight for height *z*‐score (WHZ) were calculated according to WHO growth standards. Stunting and wasting defined as HAZ < −2SD and WHZ < −2SD, respectively. Anaemia was defined according to WHO criteria for children (< 110 grams/decilitre). Vitamin A measured as serum retinol, deficiency defined as inflammation adjusted serum retinol < 0.7 umol/L. Vitamin B12 measured as serum B12, deficiency defined as < 221 pmol/L. Iron measured as serum ferritin, iron deficiency defined as inflammation adjusted serum ferritin < 12 mcg/L. Zinc measured as low serum zinc, deficiency defined as inflammation adjusted serum zinc < 65 mcg/L. Micronutrient deficiency index calculated as the mean % of deficiencies out of the number of biomarkers measured of vitamin A, vitamin B12, iron and zinc.

The average age of children in the sample was 41.2 months (Table 1). Reported morbidity was common, with approximately one‐third of the children reporting fever and/or diarrhoea in the 7 days preceding the blood draw. Reported consumption of micronutrient powders (MNP) or other vitamin mineral supplements in the previous 30 days was ≤ 8% and 24.5% reported receipt of high‐dose vitamin A capsules in the previous 6 months. Stunting and anaemia each affected approximately one‐third of children. Approximately 19% of children had low serum B12, 28% had low inflammation‐adjusted serum retinol, 58% had low inflammation‐adjusted serum ferritin and 67% had low inflammation‐adjusted serum zinc (Table 1). The average micronutrient deficiency index was 0.43 (i.e., the average proportion of micronutrient biomarker values below thresholds for deficiency or insufficiency).

### 3.2 Consumption of Fruits, Vegetables, SSB, Salty Snacks and Sweet Snacks

**Figure 1:**
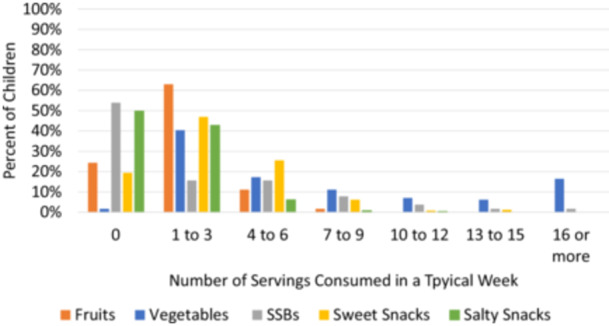
Frequency of servings of fruits, vegetables, SSBs, sweet snacks and salty snacks consumed in a typical week by children 2–5 years of age enroled in the CoMIT Pilot Survey (*n* = 243) in the Tolon and Kumbungu districts, northern Ghana. The bars represent the percent of children consuming the range of servings indicated on the x‐axis (0, 1–3, 4–6, 7–9, 10–12, 13–15 and 16 or more), for each food category. The number of servings in a typical week was calculated as reported number of days in a typical week each food was consumed multiplied by the reported number of servings consumed on a typical day. CoMIT, Condiment Micronutrient Innovation Trial; SSB, sugar‐sweetened beverages.

Approximately 76% of children had any reported fruit consumption in a typical week, and the frequency of servings consumed ranged from 1 to 3 servings in a typical week for most children (Figure 1, Tables 2, S1). Most children (98%) were reported to consume at least one serving of vegetables in a typical week, with 41% reportedly consuming ≥ 7 servings, while 17% and 40% reportedly consumed 4–6 and 1–3 servings, respectively, in a typical week (Figure 1, Tables 2, S1). The proportion of children who reached the recommendation for intake of fruits and vegetables (≥ 3 servings/day, equivalent to 250 grams/day) was 11.5% (95% CI: 7.5%–15.5%) (World Health Organization 2023). In a typical week, approximately half of the children were reported to consume SSB (46%) and salty snacks (50%), and 80% of children consumed sweet snacks. However, the frequency of salty snack and sweet snack consumption was low; most children reportedly consumed 1–3 servings in a typical week. Among children who reportedly consumed SSB, 16% consumed 1–3 servings, 16% consumed 4–6 servings and 14% consumed greater than 7 servings in a typical week (Figure 1).

**Table 2:** Reported prevalence of consumption of fruits, vegetables, SSBs and salty and sweet snacks in a typical week, among children 2–5 years of age enroled in the CoMIT pilot survey in the Tolon and Kumbungu districts, northern Ghana.^a^

	Prevalence of consumption in a typical week^a^ (*n* = 243)
Food	% (95% CIs)
Fruits	75.7 (70.3, 81.1)
Vegetables	98.4 (96.8, 99.9)
Sweet snacks	80.1 (75.7, 85.4)
Salty snacks	50.2 (43.9, 56.5)
SSBs	46.1 (39.8, 52.4)

Abbreviations: CoMIT, Condiment Micronutrient Innovation Trial; SSB, sugar‐sweetened beverages.

^a^ Consumption is defined as greater than 0 servings in a typical week for fruits, vegetables sweet snacks, salty snacks and SSBs. Because nearly all children were consuming at least one serving of vegetables in a typical week, an additional definition of consumption was calculated as ≥ 7 servings/week, as that was about the average. The prevalence of consuming ≥ 7 servings of vegetables in a typical week was 40.7 (34.6, 46.9).

### 3.3 Predictors of Consumption of Fruits, Vegetables, SSB and Sweet and Salty Snacks

Predictors of fruit, vegetable, SSB, salty snack and sweet snack consumption are reported in Table 3. Individual‐level variables found to predict consumption in at least one group were child age and sex. Older children were less likely to be consumers of fruits than younger children. Compared to females, male children were more likely to report consumption of vegetables and salty snacks. No maternal characteristics were found to predict intake.

**Table 3:** Associations between consumption^a^ of foods and individual and household characteristics among children 2–5 years of age enroled in the CoMIT Pilot Survey in the Tolon and Kumbungu districts, northern Ghana.^a^

		Minimally adjusted analysis		Fully adjusted analysis	
Predictor	Category	OR (95% CI)	*p* value	OR (95% CI)	*p* value
**Fruits (consumption > 0 servings in a typical week)**					
Child age	Continuous	0.98 (0.95, 1)	0.08	**0.97 (0.94, 1)**	**0.04**
Child sex	Male Female	1.06 (0.58, 1.93) Ref	0.86	0.92 (0.49, 1.73) Ref	0.79
District	Kumbungu Tolon	0.9 (0.49, 1.63) Ref	0.72	1.14 (0.58, 2.24) Ref	0.70
Residence area	Urban Rural	**1.94 (1.06, 3.57)** **Ref**	**0.03**	1.36 (0.66, 2.81) Ref	0.41
Household size	Continuous	1.01 (0.96, 1.07)	0.59	0.97 (0.91, 1.03)	0.28
Household food insecurity score	Continuous	**1.11 (1.02, 1.22)**	**0.02**	**1.18 (1.07, 1.3)**	**0.001**
Household asset index	Continuous	1.33 (0.98, 1.82)	0.07	**1.78 (1.2, 2.63)**	**0.004**
**Vegetables (consumption of ≥ 7 servings in a typical week)**					
Child age	Continuous	0.98 (0.96, 1.01)	0.15	0.98 (0.96, 1.01)	0.16
Child sex	Male Female	**1.97 (1.14, 3.38)** **Ref**	**0.01**	**2.01 (1.13, 3.57)** **Ref**	**0.02**
District	Kumbungu Tolon	0.81 (0.48, 1.38) Ref	0.45	0.92 (0.52, 1.63) Ref	0.77
Residence area	Urban Rural	**0.54 (0.32, 0.92)** **Ref**	**0.02**	0.55 (0.29, 1.02) Ref	0.06
Household size	Continuous	1.03 (0.99, 1.08)	0.17	1.03 (0.98, 1.09)	0.27
Household food insecurity score	Continuous	**1.16 (1.08, 1.25)**	**< 0.001**	**1.17 (1.08, 1.27)**	**< 0.001**
Household asset index	Continuous	0.93 (0.71, 1.22)	0.6	0.99 (0.71, 1.39)	0.957
**Sweet snacks (consumption > 0 servings in a typical week)**					
Child age	Continuous	0.99 (0.96, 1.02)	0.46	0.99 (0.96, 1.02)	0.439
Child sex	Male	1.16 (0.59, 2.27)	0.67	1.07 (0.52, 2.17)	0.859
	Female	Ref		Ref	
District	Kumbungu Tolon	1.25 (0.62, 2.53) Ref	0.53	1.54 (0.68, 3.48) Ref	0.298
Residence area	Urban Rural	**4.2 (1.95, 9.05)** **Ref**	**> 0.001**	**3.41 (1.39, 8.37)**	**0.008**
Household size	Continuous	1.06 (0.99, 1.13)	0.07	1.02 (0.95, 1.1)	0.533
Household food insecurity score	Continuous	**1.12 (1.01, 1.24)**	**0.03**	**1.15 (1.03, 1.29)**	**0.011**
Household asset index	Continuous	1.42 (0.99, 2.04)	0.06	1.56 (0.99, 2.44)	0.05
**Salty snacks (consumption > 0 servings in a typical week)**					
Child age	Continuous	1 (0.98, 1.03)	0.91	1 (0.98, 1.03)	0.85
Child sex	Male Female	**1.97 (1.09, 3.58)** **Ref**	**0.03**	**1.92 (1.02, 3.62)** **Ref**	**0.04**
District	Kumbungu Tolon	**0.2 (0.09, 0.45)** **Ref**	**> 0.001**	**0.22 (0.09, 0.54)** **Ref**	**0.001**
Residence area	Urban Rural	0.62 (0.28, 1.37) Ref	0.24	0.42 (0.16, 1.09) Ref	0.075
Household size	Continuous	**1.06 (1.01, 1.12)**	**0.03**	1.03 (0.97, 1.09)	0.42
Household food insecurity score	Continuous	**1.1 (1.02, 1.19)**	**0.02**	**1.15 (1.05, 1.26)**	**0.003**
Household asset index	Continuous	**1.53 (1.11, 2.11)**	**0.01**	**1.68 (1.14, 2.49)**	**0.01**
**SSB (consumption > 0 servings in a typical week)**					
Child age	Continuous	1 (0.98, 1.03)	0.67	1 (0.97, 1.03)	0.914
Child sex	Male Female	1.21 (0.72, 2.04) Ref	0.46	1.3 (0.7, 2.41) Ref	0.41
District	Kumbungu Tolon	1.07 (0.64, 1.78) Ref	0.81	1.4 (0.75, 2.62) Ref	0.286
Residence area	Urban Rural	1.5 (0.9, 2.51) Ref	0.12	1.88 (0.94, 3.73) Ref	0.072
Household size	Continuous	1.1 (1.05, 1.15)	**> 0.001**	**1.09 (1.02, 1.15)**	**0.007**
Household food insecurity score	Continuous	**1.34 (1.23, 1.47)**	**> 0.001**	**1.38 (1.25, 1.52)**	**< 0.001**
Household asset index	Continuous	1.09 (0.84, 1.41)	0.53	1.26 (0.88, 1.81)	0.212

Abbreviations: CoMIT, Condiment Micronutrient Innovation Trial; SSB, sugar‐sweetened beverages.

^a^ Consumption is defined as > 0 servings in a typical week for fruits, sweet snacks, salty snacks and SSB. For vegetables, consumption was defined as ≥ 7 or more servings in a typical week. The minimally adjusted models included district and residence as covariates, and cluster was included as a mixed effect. The fully adjusted models included child age, child sex, district, residence area, household size, household food insecurity score and household asset index. Other covariates were considered for inclusion in the fully adjusted models, however, a two‐step approach was taken to build the model and only those that were associated (*p* < 0.1) or those found to be reasonably justified to be included (child age and sex and those associated with two or more of the foods (asset index and household size) were included in the multivariable model. Covariates considered that were not included in the fully adjusted models (*p* > 0.1 in minimally adjusted models) were breastfeeding status, recent morbidity, maternal age and education. Characteristics associated with the outcome (*p* < 0.05, bolded) were considered significant predictors. For a full description of methods, including a full list of covariates considered, please see the SAP (https://osf.io/t3zrn/).

Household‐level variables found to predict intake in at least one group were district, residence area, household size, household food insecurity score and asset index; food insecurity was the only consistent predictor across all food categories.

Children in rural areas were more likely to report consumption of fruits, vegetables and sweet snacks than children in urban areas in the minimally adjusted model; however, in the fully adjusted model this relationship only remained significant for sweet snacks. Children from households in Tolon were less likely to report consumption of salty snacks than children in Kumbungu. Children in larger households were more likely to report consumption of salty snacks and SSBs, however the results only remained significant for SSBs (1.09 [1.02, 1.15], *p* = 0.01) after adjustment for the covariates. Children in households with a higher asset index were more likely to report consumption of fruits and salty snacks. Children in households with higher food insecurity scores were more likely to report consumption of foods in all categories than children in households with lower food insecurity scores; these results remained significant in the fully adjusted models. Given the counterintuitive association of reported food consumption with food insecurity, we conducted exploratory analyses in which we assessed the associations between food insecurity and continuous outcome variables (servings consumed/week), as well as associations between categorical (four‐level) and binary food insecurity variables and outcome variables. The results were consistent with those of the a priori analyses.

### 3.4 Dietary Predictors of Nutritional Status

Consumption of the selected food categories was not associated with measures of nutritional status, with two exceptions (Tables 4, S2): reported consumers of fruit had a 0.42 SD higher WHZ score than non‐consumers of fruit and reported consumers of ≥ 7 servings of vegetables in a typical week had a 4.9 g/dL higher haemoglobin concentration than consumers of < 7 servings of vegetables in a typical week. However, the same relationships were not observed in the corresponding categorical variables of wasting and anaemia. Salty snack consumption was positively associated with micronutrient index in the minimally adjusted model, where salty snack consumers had a 0.095 (~9.5%) higher micronutrient deficiency index than non‐salty snack consumers; however, the relationship was no longer significant in the fully adjusted model.

**Table 4:** Associations between consumption of foods and individual nutritional status of children 2–5 years of age enroled in the CoMIT Pilot Survey in the Tolon and Kumbungu Districts, northern Ghana.

		Minimally adjusted analysis^b^		Fully adjusted analysis^b^	
Predictor	Category	OR (95% CI)	*p* value	OR (95% CI)	*p* value
**Stunting (HAZ < −2)** a					
Fruits	Consumer Non‐consumer	1.18 (0.61, 2.28) Ref	0.62	1.57 (0.74, 3.34) Ref	0.24
Vegetables	Consumer Non‐consumer	0.65 (0.36, 1.19) Ref	0.16	0.64 (0.33, 1.24) Ref	0.18
Sweet snacks	Consumer Non‐consumer	0.57 (0.29, 1.13) Ref	0.11	0.55 (0.25, 1.21) Ref	0.14
Salty snacks	Consumer Non‐consumer	0.57 (0.31, 1.05) Ref	0.07	0.5 0.25, 1.02) Ref	0.06
SSBs	Consumer Non‐consumer	1.18 0.67, 2.08) Ref	0.57	1.98 (0.95, 4.15) Ref	0.07
Child age	Continuous	1.01 (0.98, 1.03)	0.62	1.01 (0.98, 1.04)	0.55
Child sex	Male Female	0.93 (0.53, 1.64) Ref	0.79	1.17 (0.64, 2.14) Ref	0.62
Reported recent morbidity	Yes No	1.74 (0.97, 3.13) Ref	0.07	**1.95 (1.05, 3.61)** **Ref**	**0.04**
Household food insecurity score	Continuous	0.98 (0.91, 1.06)	0.63	0.97 (0.88, 1.06)	0.45
District	Kumbungu Tolon	1.05 (0.6, 1.84) Ref	0.87	0.8 (0.42, 1.52) Ref	0.50
Residence area	Urban Rural	**0.49 (0.28, 0.87)** **Ref**	**0.02**	**0.41 (0.22, 0.8)** **Ref**	**0.01**
**Wasting (WHZ < −2)** a					
Fruits	Consumer Non‐consumer	0.43 (0.12, 1.51) Ref	0.19	0.35 (0.07, 1.62) Ref	0.18
Vegetables	Consumer Non‐consumer	1.08 (0.31, 3.75) Ref	0.9	1.66 (0.36, 7.7) Ref	0.51
Sweet snacks	Consumer Non‐consumer	0.86 (0.21, 3.57) Ref	0.84	0.88 (0.17, 4.5) Ref	0.88
Salty snacks	Consumer Non‐consumer	0.67 (0.18, 2.4) Ref	0.53	0.74 (0.17, 3.25) Ref	0.69
SSBs	Consumer Non‐consumer	0.74 (0.21, 2.66) Ref	0.65	0.89 (0.18, 4.54) Ref	0.89
Child age	Continuous	0.98 (0.93, 1.04)	0.53	0.98 (0.92, 1.04)	0.44
Child sex	Male Female	1.21 (0.35, 4.16) Ref	0.76	1.03 (0.27, 3.86) Ref	0.97
Household asset index	Continuous	2 (0.98, 4.06)	0.06	**2.15 (1.03, 4.49)**	**0.04**
District	Kumbungu Tolon	0.87 (0.26, 2.99) Ref	0.83	0.99 (0.25, 3.92) Ref	0.99
Residence area	Urban Rural	0.37 (0.1, 1.46) Ref	0.16	**0.2 (0.03, 1.18)** **Ref**	**0.07**
**Anaemia (< 110 g/dL)** a					
Fruits	Consumer Non‐consumer	0.6 (0.32, 1.11) Ref	0.1	0.57 (0.28, 1.16) Ref	0.12
Vegetables	Consumer Non‐consumer	0.68 (0.38, 1.2) Ref	0.18	0.64 (0.34, 1.22) Ref	0.18
Sweet snacks	Consumer Non‐consumer	0.55 (0.27, 1.14) Ref	0.11	0.63 (0.28, 1.43) Ref	0.27
Salty snacks	Consumer Non‐consumer	0.85 (0.47, 1.53) Ref	0.58	1.02 (0.51, 2.02) Ref	0.97
SSBs	Consumer Non‐consumer	0.97 (0.56, 1.68) Ref	0.92	1.54 (0.8, 2.96) Ref	0.2
Child age	Continuous	0.97 (0.95, 1)	0.03	**0.97 (0.95, 1)**	**0.03**
Child sex	Male Female	1.47 (0.85, 2.55) Ref	0.17	1.7 (0.93, 3.1) Ref	0.08
Malaria	Positive RDT Negative RDT	1.86 (0.98, 3.55) Ref	0.06	2 (0.99, 4.03) Ref	0.05
District	Kumbungu Tolon	1.22 (0.71, 2.1) Ref	0.47	1.31 (0.7, 2.47) Ref	0.4
Residence area	Urban Rural	**1.93 (1.12, 3.33)** **Ref**	**0.02**	**2.26 (1.19, 4.29)** **Ref**	**0.01**
**Micronutrient Deficiency Index (Micronutrient Index > 0.75)** a					
Fruits	Consumer Non‐consumer	1.35 (0.51, 3.54)	0.54	0.42 (0.13, 1.39) Ref	0.15
Vegetables	Consumer Non‐consumer	0.63 (0.26, 1.55)	0.31	1.08 (0.39, 2.99) Ref	0.88
Sweet snacks	Consumer Non‐consumer	0.23 (0.05, 1.08)	0.06	4.96 (0.89, 27.56) Ref	0.07
Salty snacks	Consumer Non‐consumer	0.42 (0.16, 1.1)	0.08	1.8 (0.6, 5.36) Ref	0.29
SSBs	Consumer Non‐consumer	0.69 (0.29, 1.67)	0.41	1.29 (0.45, 3.65) Ref	0.63
Child age	Continuous	1.03 (0.98, 1.07)	0.22	0.97 (0.93, 1.02)	0.3
Child sex	Male Female	**0.34 (0.13, 0.88)**	**0.03**	2.72 (0.97, 7.65) Ref	0.06
Malaria	Positive RDT Negative RDT	0.9 (0.31, 2.65)	0.85	1.69 (0.52, 5.51) Ref	0.38
District	Kumbungu Tolon	**0.34 (0.14, 0.86)**	**0.02**	**4.64 (1.54, 14.03)** **Ref**	**0.01**
Rural	Urban Rural	0.97 (0.4, 2.37)	0.95	0.75 (0.26, 2.16) Ref	0.59

Abbreviations: CoMIT, Condiment Micronutrient Innovation Trial; SSB, sugar‐sweetened beverages.

^a^ Height for age *z*‐score (HAZ) and weight for height *z*‐score (WHZ) were calculated according to WHO growth standards. Haemoglobin was measured using Hemocue 301 and anaemia was defined according to WHO criteria for children (< 110 g/dL, site reference here). Malaria was measured with a Malaria Rapid Diagnostic Test. The micronutrient deficiency index was constructed by summing up the number of micronutrient deficiencies among iron (serum ferritin), zinc (serum zinc), vitamin B12 (serum B12) and vitamin A (serum retinol) and dividing that by the total number of micronutrient indicators measured. The micronutrient index categorical variable was created using a cut point that identified the lowest quintile of the index, which was equal to a micronutrient deficiency index of 0.75.

^b^ The minimally adjusted models included district and residence as covariates, and cluster was included as a mixed effect. The fully adjusted models included the variables listed as predictors for each of the outcomes. Other covariates were considered for inclusion in the fully adjusted models, however, a two‐step approach was taken to build the model and only those that were associated (*p* < 0.1) or those found to be reasonably justified to be included (child age and sex and if a potential covariate was associated with either the categorical or continuous outcome the covariate was included in the fully adjusted multivariable models for both versions of that outcome) were included in the multivariable model. Covariates considered but not included in any of the multi‐variable models (*p* > 0.1 in minimally adjusted models) were breastfeeding status, micronutrient powder consumption in the previous 30 days, other vitamin and mineral supplements in previous 30 days and high‐dose vitamin A supplementation in previous 6 months, maternal age and maternal education. Characteristics associated with the outcome (*p* < 0.05, bolded) were considered significant predictors. For a full description of methods, including a full list of covariates considered, please see the SAP (https://osf.io/t3zrn/).

## 4 Discussion

Descriptive information on the consumption of healthy and unhealthy food groups by young children, especially in contexts undergoing a nutrition transition, can identify where interventions are needed and guide their design. We found that the diets of children 2–5 years in northern Ghana were severely inadequate with respect to the recommended consumption of fruits and vegetables. While 98% of children consumed at least one serving of vegetables in a typical week, only ~11% reached the World Health Organization fruit and vegetable intake recommendations for children (at least 250 g or ~3 servings per day) (World Health Organization 2023). On the other hand, half of the children reported consumption of SSB in a typical week, and 87% consumed sweet or salty snacks, but average servings consumed in a typical week were low, ranging from ~1.2 servings in a typical week for salty snacks to 2.6 and 2.9 servings of SSB and sweet snacks, respectively. While there are no specific intake guidelines for SSB and unhealthy snack foods (high in sugar, fat and salt) for children 2–5 years of age, consumption of these products by young children is discouraged by the World Health Organization, and the 2009 and 2023 Ghana Dietary Guidelines for children above 5 years of age (Ministry of Food and Agriculture 2023; Ministry of Health 2010; World Health Organization 2023).

These findings align with studies among other populations in West Africa and Ghana, indicating that children are not achieving recommended consumption of fruits and vegetables or minimum dietary diversity, while SSB and unhealthy snack food availability and consumption are prevalent (C. K. Allen et al. 2023; Development Initiatives 2022; Saaka et al. 2016). A recent meta‐analysis of DHS data found that 56.1% of children 6–23 months in West and Central Africa had consumed no fruits or vegetables in the previous day (C. K. Allen et al. 2023). The 2022 Global Nutrition Report noted that in Ghana in 2017, only 26% of children 6–23 months achieved minimum dietary diversity (Development Initiatives 2022), and a study conducted in the Northern Region of Ghana found that only 34% of children 6–23 months achieved minimum dietary diversity (Saaka et al. 2016). Processed foods, including ultra‐processed foods, and SSB are widely available in urban areas of Ghana (Adjei et al. 2022; Green et al. 2020; Holdsworth et al. 2020; Mockshell et al. 2022), and while there have been increases in per capita availability of fruits and vegetables from 2010 to 2020 (Reardon et al. 2024), evidence suggests the supplies remain too low for adequate consumption of fruits and vegetables (Reardon et al. 2024). Data from the Global Diet Quality Project in Ghana found that 40% of urban adults consumed sweets and 21% consumed soft drinks on the previous day (Global Diet Quality Project 2021). While results of the reported studies are not directly comparable to our study due to the differences in recall period (previous day vs. typical week), age (children 6–23 months and adults rather than children 24–59 months) and study location (focused on urban areas) of the selected populations, the studies are consistent in demonstrating the need to increase consumption of fruits and vegetables while also minimising consumption of SSB and unhealthy snack foods in this context. In March of 2023, Ghana instituted a taxation of 20% on SSB (flavoured juice drinks, sweetened tea, soft drinks and energy drinks) to address the high sugar intake in the country (International Development Research Center 2023; Singh et al. 2023). Taxes on SSB may have the potential to reduce purchasing and consumption of these products (Bercholz et al. 2022; Eykelenboom et al. 2022), however follow‐up studies are needed to evaluate the impact of this tax on SSB purchasing patterns and intake in Ghana. Additional investments in policies or interventions to improve fruit and vegetable consumption, such as home garden programmes or national‐level agricultural programmes to increase the affordability and availability of fruits and vegetables, may be warranted. Additional data on food source (purchased vs. prepared at home), purchasing location and factors influencing consumption could guide intervention design.

There were a few household and individual factors significantly associated with consumption of the food categories examined. In general, the limited associations could be explained by the ubiquity of specific common food items in each group (e.g., tomatoes/onions) or other unmeasured factors like maternal occupation, parity and nutrition knowledge, which have been found to be associated in other studies (Agyemang et al. 2023; C. K. Allen et al. 2023; Belay et al. 2022; Kang et al. 2023; Solomon et al. 2017). We did find significant associations between consumption patterns and household asset index and household food insecurity. Children in households with a higher asset index were more likely to consume fruits, salty snacks and sweet snacks (*p* = 0.05). These findings are consistent with other studies which have found that household wealth is associated with numerous indicators of diet quality among children, specifically children in wealthier households are more likely to be consumers of fruits and vegetables and have higher minimum adequate diet and minimum dietary diversity (C. K. Allen et al. 2023; Anane et al. 2021; Belay et al. 2022; Haque et al. 2024; Kang et al. 2023; Solomon et al. 2017).

However, household food insecurity was associated with greater consumption of all food categories in an unexpected direction. Results indicated that children in households with higher food insecurity scores were more likely to be consumers of all food categories. This finding is not consistent with the existing literature, which has typically shown that children have lower diet diversity in households with higher food insecurity (Antwi et al. 2022; Roba et al. 2024). Unfortunately, we did not measure diet diversity and cannot directly compare our findings to the existing literature, but there are a few potential factors that could have contributed to these seemingly counterintuitive results. First, due to the large and often complex nature of the households in this region (13 members on average and often with multiple generations and/or polygamous marriage) and that the respondent of the food insecurity questionnaire was not necessarily the caregiver of the child, it is possible that a household‐level food insecurity questionnaire may not be an accurate proxy for food availability and access of the index child in this context. Second, households may be engaging in food insecurity coping strategies. A 2016 study conducted in and around Tamale, Ghana, identified adults restricting meals and foraging for wild foods as frequent coping strategies to food insecurity (Chagomoka et al. 2016). Convenience may also be a driver, for example, purchasing an SSB or snack food may assuage a child's hunger without necessitating meal preparation when resources are limited. Additional qualitative and quantitative research (e.g., on food insecurity coping mechanisms) may help to better understand the relationships between household‐level food insecurity and food insecurity among children, and between food security and eating behaviours and dietary patterns in this context.

We identified a few associations between consumption of selected food categories and nutritional status. This could reflect the limited information captured by the dietary indicators included in this study or the influence of unmeasured factors such as morbidity. Assessment of other dimensions of diet quality, such as dietary diversity, consumption of animal source foods, or collection of detailed quantitative data on consumption may better explain relationships between diet and nutritional status in this context. The use of a categorical consumption variable rather than a continuous estimate of servings consumed can decrease statistical power to detect associations, and may explain some of the limited associations observed. However, frequency of reported consumption of all food groups was low with little variability among the sample (among consumers, most were consuming 1–3 servings or 1–6 servings in a typical week), so it is unlikely that using a continuous outcome would have significantly changed the observed results. The use of a micronutrient index as an indicator of micronutrient adequacy is a cruder measure than using individual micronutrient biomarkers, however it allowed us to examine micronutrient adequacy through the lens of multiple deficiencies. The positive association between vegetable consumption and haemoglobin is consistent with other studies among children, which have found that vegetable and fruit intake, and higher dietary diversity, are associated with lower odds of anaemia (Augusto et al. 2015; Visser et al. 2021), potentially reflecting the nutrient density of the diet. It seems unlikely that the positive association between fruit consumption and WHZ indicates a specific role for fruit, but it is possible that this association reflects other unmeasured relationships, i.e., that fruit consumption may reflect other dimensions of resources and/or care practices that were not captured in our measures of assets.

Dietary assessment is a challenge, particularly for young children. We chose a simplified diet questionnaire to minimise participant burden and chose to adapt the WHO STEPS questionnaire as there were no validated tools for assessing both healthy and unhealthy food groups among children 2–5 years of age in this context at the time of the study. The adapted tool we used was not formally validated for use among children 2–5, which was a limitation. Assessment of intake in our study could have been impacted by typical limitations of dietary assessments, including social desirability bias, recall bias and challenges in estimating quantities consumed (Bailey 2021). Furthermore, estimation of child intake is particularly difficult in settings such as this, where there is the potential for multiple caregivers to feed the child and where multiple children are fed from the same pot of food. The questionnaire respondent was the primary caregiver of the child (87% reported to be the child's mother), but we did not collect data on whether the child was fed by other caregivers or members of the household. In addition, due to the cross‐sectional design, intake over different seasons could not be captured, and fruit and vegetable intake could vary greatly depending on the season captured. Lastly, while we asked for estimates of servings consumed on a ‘typical day’, we did not have a quantitative measure of consumption, which may better explain relationships between consumption of specific food categories and health risk.

Despite these limitations, our results provide insight into fruit, vegetable, SSB and snack food consumption among pre‐school age children in the context of a population undergoing a nutrition transition coupled with high rates of undernutrition, food insecurity and poverty (Ghana Statistical Survey & UNICEF 2019). IYCF indicators for children 6–23 months have been widely adopted in surveys such as DHS; however, less information is available on the diets of children 2–5 years of age in LMICs. In Ghana, information on the consumption of SSB and snack foods among this age group and outside of urban centres is lacking. Nutrition remains critical to growth and development beyond the first 24 months of life and understanding children's dietary patterns is important for identifying areas of public health concern that may be addressed by policies or interventions.

In conclusion, we found that reported dietary intake among children 2–5 years in northern Ghana was severely inadequate with respect to fruit and vegetable consumption, while SSB and salty and sweet snack consumption were common. Interventions or national policies to increase fruit and vegetable consumption while minimising SSB and related products are needed across all subgroups of children 2–5 years within this area. Further information on food source, purchasing patterns and factors driving consumption will aid in the identification and design of appropriate interventions or policies. There were limited associations found between household, maternal and individual factors and consumption of the food categories measured, and between consumption of the food categories and individual nutritional status. Research on the relationships between individual and household‐level food insecurity, including food insecurity coping mechanisms and the collection of more detailed dietary data, can be used to better understand drivers of consumption among young children in this context.

## Author Contributions

The authors' responsibilities were as follows: E.R.B., S.M.K., C.D.A., S.A.‐A. and R.E.‐S. conceptualised the research. E.R.B., S.M.K., J.N.D., C.D.A., K.R.W., X.T., A.D.F., K.P.A., M.J.H., S.A.V., S.A.‐A., R.E.‐S. developed the methodology. S.A.‐A., S.M.K., K.R.W., M.J.H., S.A.V. and R.E.‐S. were responsible for project administration. E.R.B., S.M.K., J.N.D,. A.D.F. and S.A.‐A. supervised data collection. C.D.A., X.T. and E.R.B. analysed data. E.R.B. and R.E.‐S. draughted the manuscript; and all authors reviewed and edited the manuscript. All authors read and approved the final version of the manuscript and accepted final responsibility for its content.

## Conflicts of Interest

The authors declare no conflicts of interest.

## Supporting information

Child‐Dietary‐Patterns_Online‐Supplementary‐Materials_Revised_CLEAN.

Supplemental‐File‐1_Serving‐Size‐Showcards1.

Supplemental‐File‐2_Adapted‐Diet‐Questions1.

## Data Availability

The data that support the findings of this study are available from the corresponding author upon reasonable request.
