# Supplementary material for: Consumption of Selected Healthy and Unhealthy Food Groups and Associations With Nutritional Status Among Children 2–5 Years of Age in Northern Ghana
Source: Matern Child Nutr. 2025 Nov 18;22(1):e70126. doi: 10.1111/mcn.70126 (PMC12624276; doi:10.1111/mcn.70126)
Supplement: Supplementary file 1 — Child‐Dietary‐Patterns_Online‐Supplementary‐Materials_Revised_CLEAN. [file MCN-22-e70126-s002.docx]

**Online Supplementary Materials**

**Supplemental Table 1.** Average number of servings consumed in a typical week by children 2-5 years of age enrolled in the CoMIT Pilot Survey in the Tolon and Kumbungu Districts, northern Ghana^1^

| **Food Category** | **Mean (Std)** |
| --- | --- |
| Fruits | 1.80 (1.68) |
| Vegetables | 7.86 (7.26) |
| SSB | 2.63 (3.85) |
| Sweet Snacks | 2.86 (2.54) |
| Salty Snacks | 1.16 (1.68) |

^1^The number of servings in a typical week was calculated as reported number of days in a typical week each food was consumed multiplied by the reported number of servings consumed on a typical day. Abbreviations: CoMIT, Condiment Micronutrient Innovation Trial

**Supplemental Table 2.**  Prevalence of children 2-5 years of age enrolled in the CoMIT Pilot Survey in the Tolon and Kumbungu Districts, northern Ghana who reach the World Health Organization recommendations for servings of fruits and vegetables on a typical day.

| **Food Category** | **Prevalence meeting WHO recommendation^1^**  **% (95% CI)** |
| --- | --- |
| Fruits and Vegetables | 11.5 (7.5, 15.5) |

^1^WHO recommendation is 250g/day or ~3 servings/day for fruits and vegetables combined.

Abbreviations: CoMIT; Condiment Micronutrient Innovation Trial.

**Supplemental Table 3.** Associations between consumption of foods and continuous indicators of individual nutritional status of children 2-5 years of age enrolled in the CoMIT Pilot Survey in the Tolon and Kumbungu Districts, northern Ghana^1^

|  | | **HAZ (continuous)^1^** | | | |
| --- | --- | --- | --- | --- | --- |
|  |  | **Minimally Adjusted Analysis** | | **Fully Adjusted Analysis** | |
| **Predictor** | **Category** | **OR (95% CI)** | **P** | **OR (95% CI)** | **P** |
| Fruits | Consumer  Non-consumer | 0.09(-0.35, 0.54)  Ref | 0.68 | 0.07(-0.42, 0.55)  Ref | 0.79 |
| Vegetables | Consumer  Non-consumer | 0.01(-0.38, 0.41)  Ref | 0.94 | 0.09(-0.35, 0.52)  Ref | 0.70 |
| Sweet Snacks | Consumer  Non-consumer | 0.11(-0.39, 0.61)  Ref | 0.67 | 0.11(-0.43, 0.65)  Ref | 0.69 |
| Salty Snacks | Consumer  Non-consumer | 0.12(-0.3, 0.53)  Ref | 0.58 | 0.26(-0.2, 0.72)  Ref | 0.27 |
| SSBs | Consumer  Non-consumer | -0.26(-0.65, 0.13)  Ref | 0.19 | -0.26(-0.75, 0.22)  Ref | 0.28 |
| Child age | Continuous | **-0.02(-0.04, -0.01)** | **0.01** | **-0.02(-0.04, -0.01)** | **0.01** |
| Child sex | Male  Female | 0.03(-0.36, 0.42)  Ref | 0.89 | -0.05(-0.45, 0.35)  Ref | 0.82 |
| Reported Recent Morbidity | Yes  No | -0.23(-0.64, 0.18)  Ref | 0.28 | -0.26(-0.68, 0.15)  Ref | 0.21 |
| Household food insecurity | Continuous | -0.05(-0.1, 0) | 0.07 | -0.04(-0.1, 0.02) | 0.18 |
| District | Kumbungu  Tolon | 0.28(-0.11, 0.67)  Ref | 0.15 | 0.33(-0.09, 0.75)  Ref | 0.13 |
| Residence area | Urban  Rural | 0.39(0, 0.78)  Ref | 0.05 | 0.37(-0.06, 0.79)  Ref | 0.09 |
|  |  | **WHZ (continuous)^1^** | | | |
| Fruits | Consumer  Non-consumer | **0.31(0.03, 0.58)**  **Ref** | **0.03** | **0.42(0.12, 0.72)**  **Ref** | **0.01** |
| Vegetables | Consumer  Non-consumer | -0.01(-0.25, 0.24)  Ref | 0.95 | -0.05(-0.31, 0.21)  Ref | 0.71 |
| Sweet Snacks | Consumer  Non-consumer | 0.06(-0.25, 0.37)  Ref | 0.71 | 0.08(-0.26, 0.41)  Ref | 0.64 |
| Salty Snacks | Consumer  Non-consumer | -0.1(-0.36, 0.16)  Ref | 0.45 | -0.1(-0.39, 0.18)  Ref | 0.48 |
| SSBs | Consumer  Non-consumer | -0.08(-0.32, 0.16)  Ref | 0.51 | -0.16(-0.43, 0.11)  Ref | 0.26 |
| Child age | Continuous | 0(-0.01, 0.01) | 0.83 | 0(-0.01, 0.01) | 0.83 |
| Child sex | Male  Female | 0(-0.24, 0.24)  Ref | 0.99 | 0.04(-0.21, 0.28)  Ref | 0.76 |
| Asset Index | Continuous | -0.11(-0.23, 0.01) | 0.07 | **-0.12(-0.25, 0)** | **0.05** |
| District | Kumbungu  Tolon | 0.17(-0.08, 0.43)  Ref | 0.18 | 0.12(-0.16, 0.4)  Ref | 0.40 |
| Residence area | Urban  Rural | -0.03(-0.28, 0.23)  Ref | 0.84 | 0.02(-0.28, 0.32)  Ref | 0.90 |
|  |  | **Hemoglobin (continuous)** | | | |
| Fruits | Consumer  Non-consumer | 2.19(-2.38, 6.77)  Ref | 0.35 | 2.16(-2.8, 7.12)  Ref | 0.39 |
| Vegetables | Consumer  Non-consumer | **4.2(0.14, 8.25)**  **Ref** | **0.04** | **4.85(0.52, 9.18)**  **Ref** | **0.03** |
| Sweet Snacks | Consumer  Non-consumer | 3.92(-1.28, 9.11)  Ref | 0.14 | 2.74(-2.89, 8.37)  Ref | 0.34 |
| Salty Snacks | Consumer  Non-consumer | 0.85(-3.42, 5.12)  Ref | 0.69 | -0.54(-5.16, 4.08)  Ref | 0.82 |
| SSBs | Consumer  Non-consumer | 0.58(-3.42, 4.57)  Ref | 0.78 | -2.3(-6.75, 2.16)  Ref | 0.31 |
| Child Age | Continuous | **0.24(0.07, 0.42)** | **0.01** | **0.26(0.09, 0.44)** | **0.003** |
| Child Sex | Male  Female | -1.97(-5.94, 2.01)  Ref | 0.33 | -2.75(-6.79, 1.3)  Ref | 0.18 |
| Malaria | Positive RDT  Negative RDT | -4.15(-8.84, 0.53)  Ref | 0.08 | -3.87(-8.65, 0.9)  Ref | 0.11 |
| District | Kumbungu  Tolon | -1.27(-5.22, 2.67)  Ref | 0.53 | -1.71(-5.96, 2.54)  Ref | 0.43 |
| Residence area | Urban  Rural | **-4.72(-8.66, -0.77)**  **Ref** | **0.02** | **-4.96(-9.24, -0.68)**  **Ref** | **0.02** |
|  |  | **Micronutrient Deficiency Index (continuous)^1^** | | | |
| Fruits | Consumer  Non-consumer | -3.84(-12.59, 4.9)  Ref | 0.39 | -7.97(-17.41, 1.47)  Ref | 0.1 |
| Vegetables | Consumer  Non-consumer | 3.35(-4.3, 11)  Ref | 0.39 | 0.01(-8.19, 8.2)  Ref | 1 |
| Sweet Snacks | Consumer  Non-consumer | 5.13(-4.24, 14.5)  Ref | 0.28 | 4.05(-6.37, 14.47)  Ref | 0.44 |
| Salty Snacks | Consumer  Non-consumer | **9.78(1.8, 17.76)**  **Ref** | **0.02** | 8.57(-0.21, 17.35)  Ref | 0.06 |
| SSBs | Consumer  Non-consumer | 2.24(-5.4, 9.88)  Ref | 0.56 | 0.55(-8.11, 9.21)  Ref | 0.9 |
| Child Age | Continuous | -0.33(-0.68, 0.02) | 0.07 | -0.35(-0.72, 0.01) | 0.06 |
| Child Sex | Male  Female | **11.02(3.58, 18.47)**  **Ref** | **0.004** | **8.75(0.87, 16.63)**  **Ref** | **0.03** |
| Malaria | Positive RDT  Negative RDT | -3.18(-12.36, 6.01)  Ref | 0.49 | 0.74(-8.48, 9.95)  Ref | 0.87 |
| District | Kumbungu  Tolon | **9.2(0.34, 18.06)**  **Ref** | **0.04** | **13.18(3.68, 22.68)**  **Ref** | **0.01** |
| Rural | Urban  Rural | -1.53(-10.38, 7.32)  Ref | 0.73 | -2.49(-11.96, 6.97)  Ref | 0.6 |

^1^Height for age z-score (HAZ) and weight for height z-score (WHZ) were calculated according to WHO growth standards. Hemoglobin was measured using Hemocue 301 and anemia was defined according to WHO criteria for children (<110 g/dL, site reference here). Malaria was measured with a Malaria Rapid Diagnostic Test. The micronutrient deficiency index was constructed by summing up the number of micronutrient deficiencies among iron (serum ferritin), zinc (serum zinc), vitamin B12 (serum B12) and vitamin A (serum retinol) and dividing that by the total number of micronutrient indicators measured. The micronutrient index categorical variable was created using a cut point that identified the lowest quintile of the index. Abbreviations: CoMIT, Condiment Micronutrient Innovation Trial; HAZ, height for age z-score; WHZ, weight for height z-score.

**
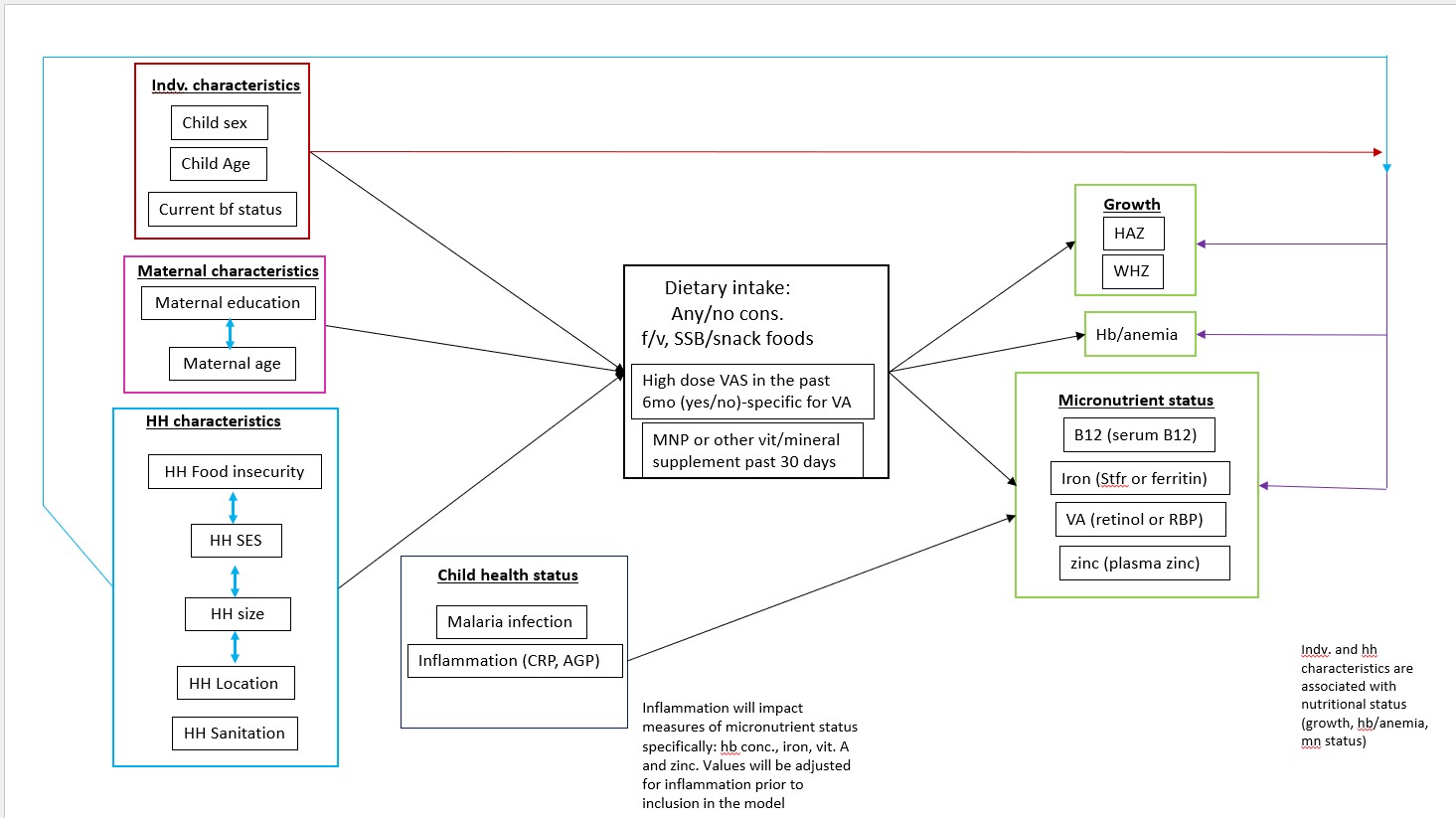
**

**Supplemental Figure 1.** Conceptual model of hypothetical relationships between household, maternal and individual factors and dietary intake and the relationships between dietary intake and nutritional status.

**Supplemental Figure 1 Legend.** A conceptual model created and used to select potential covariates for the multivariable models to 1) identify household, maternal and individual predictors of child dietary intake and 2) identify what components of diet were related to nutritional status of children. Abbreviations: bf, breastfeeding; HH, household; SES, socioeconomic status; CRP, c-reactive protein; AGP, alpha-1-acid glycoprotein; cons., consumption; f/v, fruits/vegetables; VAS, vitamin A supplementation; hb, hemoglobin; HAZ, height-for-age z-score; WHZ, weight-for-height z-score; Stfr, serum transferrin receptor; VA, vitamin A.

**
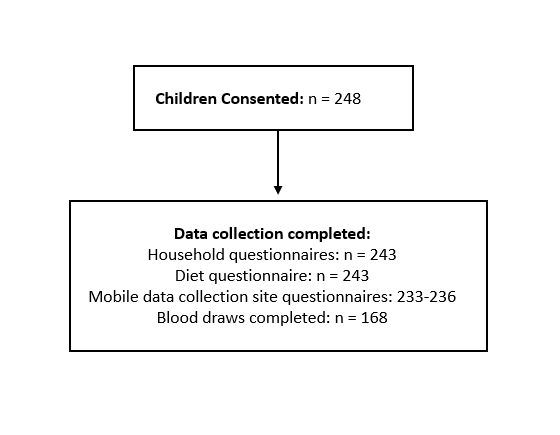
**

**Supplemental Figure 2.** Diagram of data collected from children (their caregivers) enrolled in the CoMIT Pilot Survey.

**Supplemental Figure 2 Legend.** Household questionnaires included the household roster, household assets and household food insecurity questionnaire. Not all children consented completed the study. Not all children that went to the mobile data collection site had all data collected, therefore there is a range of questionnaires completed rather than one number representing the different activities at the site (biospecimen questionnaire, anthropometry questionnaire, rapid diagnostics questionnaire including malaria RDT and hemoglobin).
