# Supplementary material for: Consumption of Selected Healthy and Unhealthy Food Groups and Associations With Nutritional Status Among Children 2–5 Years of Age in Northern Ghana
Source: Matern Child Nutr. 2025 Nov 18;22(1):e70126. doi: 10.1111/mcn.70126 (PMC12624276; doi:10.1111/mcn.70126)
Supplement: Supplementary file 2 — Supplemental‐File‐1_Serving‐Size‐Showcards1. [file MCN-22-e70126-s001.pdf]

Adult and child serving sizes for fruits and vegetables

- **A standard serving size is 80 g.**
- **80 g = 1 piece of fruit or vegetable the size of a tennis ball**
- **80 g = 2 pieces of fruit or vegetable each the size of an egg**
- **80 g = a handful of small fruits or vegetables**

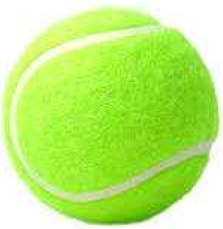

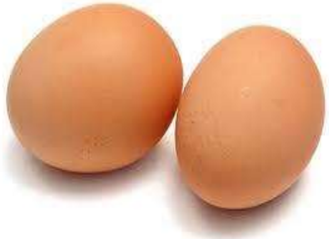

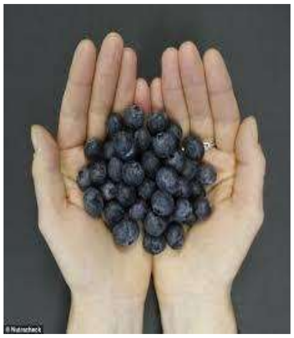

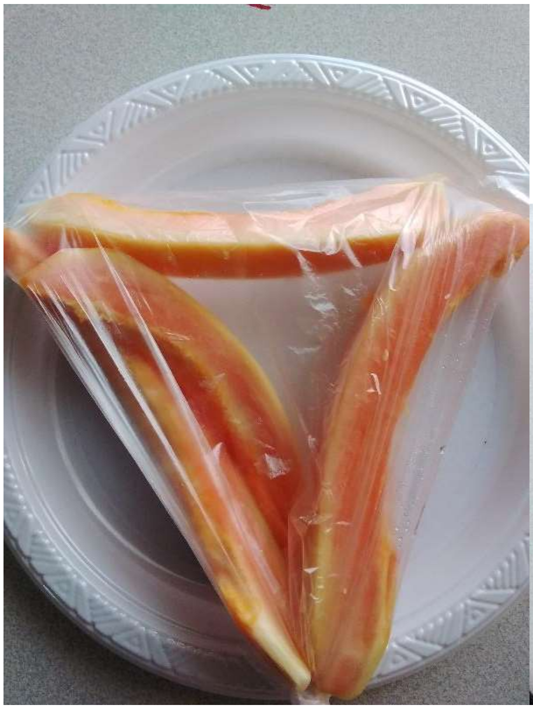

Papaya = ~100 g or 1.25 servings

Watermelon = ~450 g or 5.5 servings

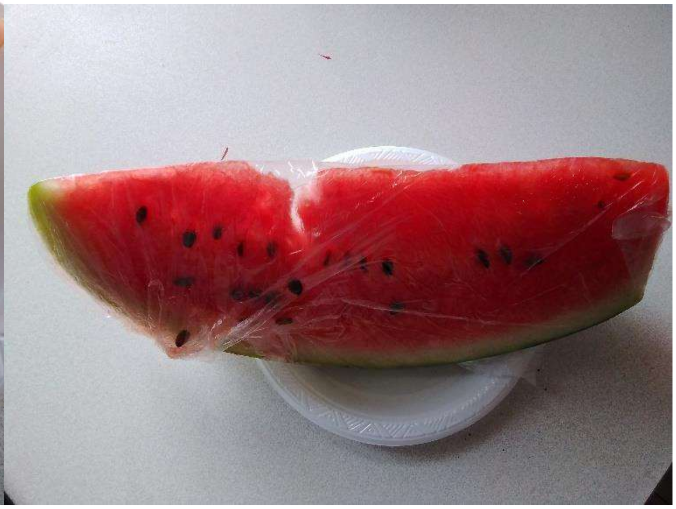

Note: Fruit or vegetable juice, or canned fruit should not be included. Tubers such as potatoes and cassava should not be included.

---

1 standard serving of cooked vegetables is 80g.

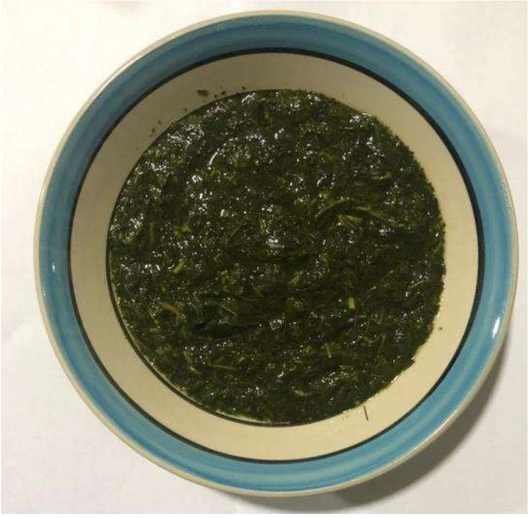

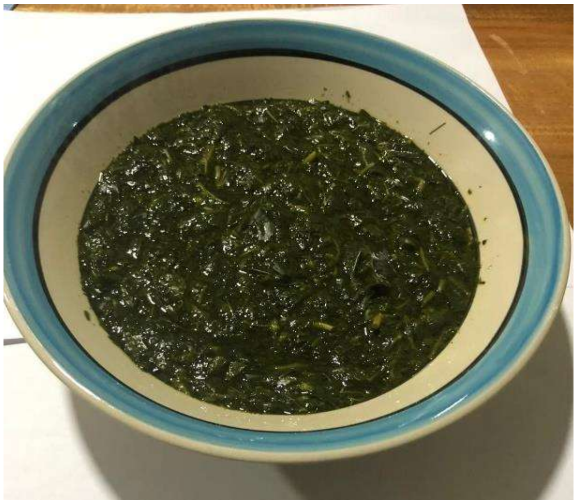

320 g cooked vegetables = 4 servings

Servings of sweet snacks, such as candies, sweet biscuits, and chocolates

- Adult serving size: 30 g for biscuits or chocolates, 15 g for candies (2 candies)
- Child serving size: 20 g for biscuits or chocoloates, 7 g for candies (1 candy)

20 g **30 g**

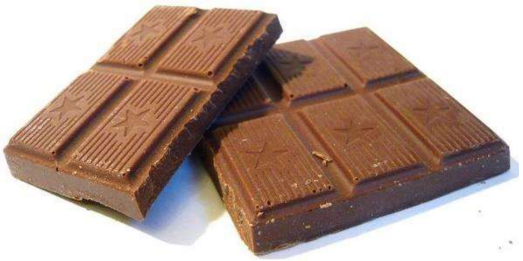

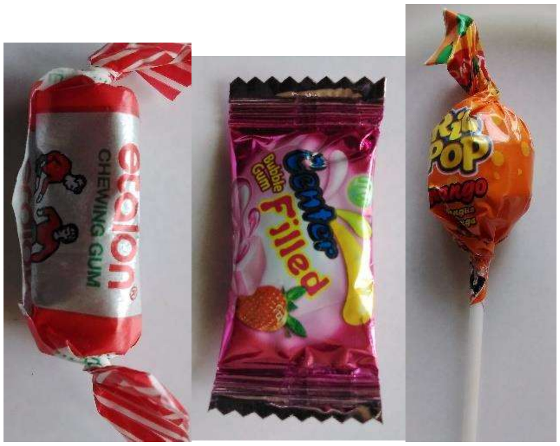

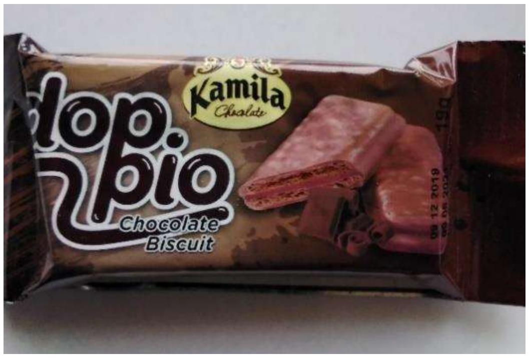

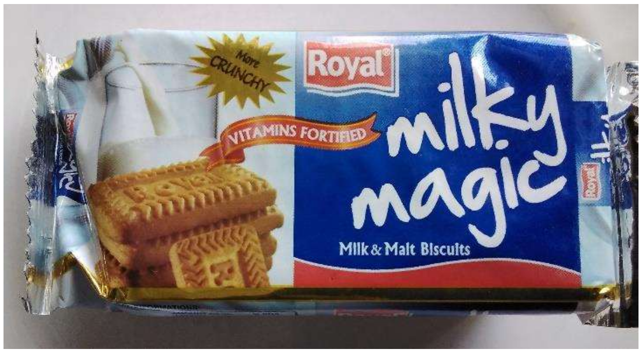

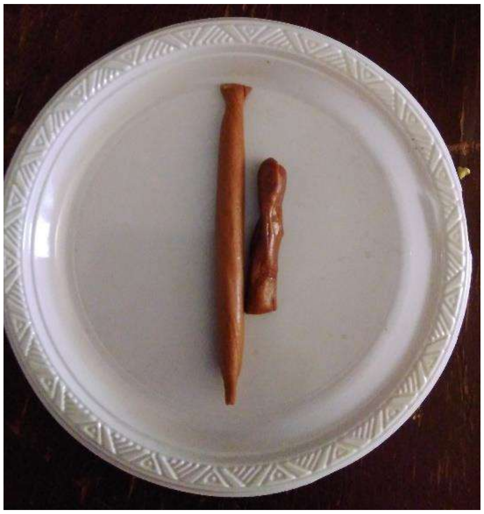

Condensed milk toffee candy
Long = 13 g = 1 adult serving
Short = 6 g = 1 child serving

Servings of sugar-sweetened beverages (any beverage where sugar was added by the manufacturer or by the consumer)

- Adult serving size: 360 mL
- Child serving size: 120 mL

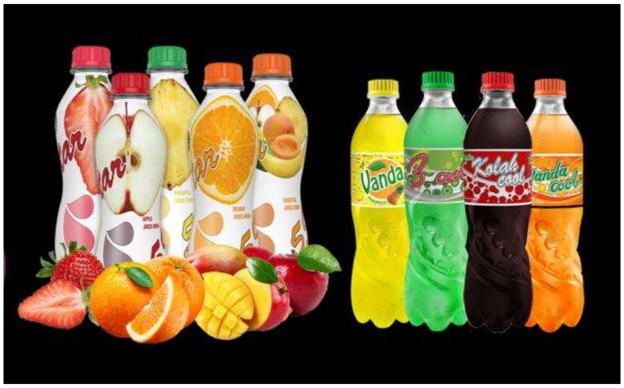

= 1 adult serving or 2 child servings

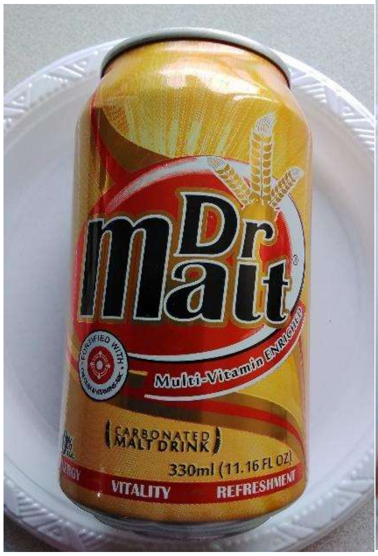

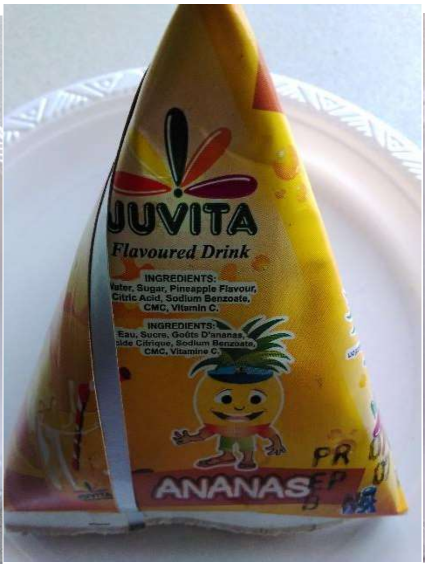

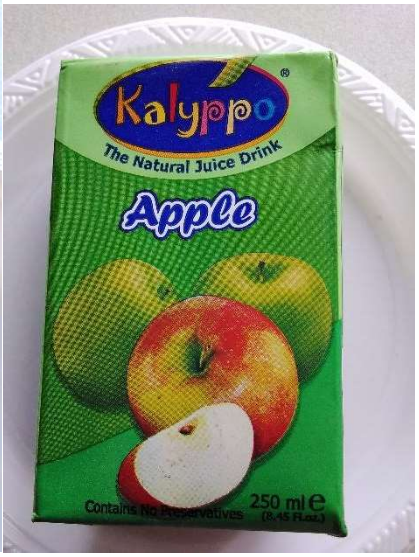

= 1 adult serving

= 1.5 child servings

= 2 child servings

(This juice is sweetened with sugar)

Other sweetened beverages:

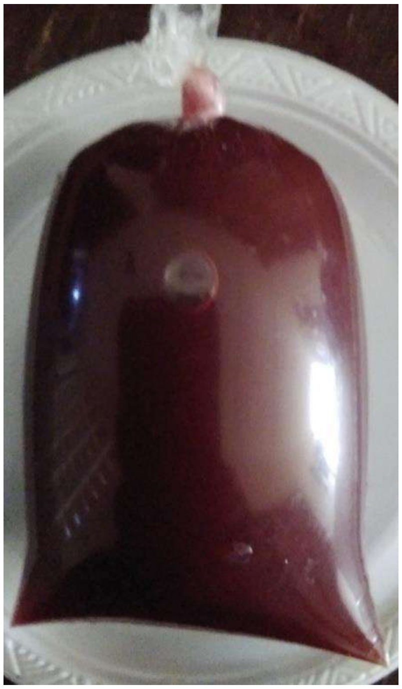

Sobolo (hibiscus) drink = 370 mL
= 1 adult serving
= 3 child servings

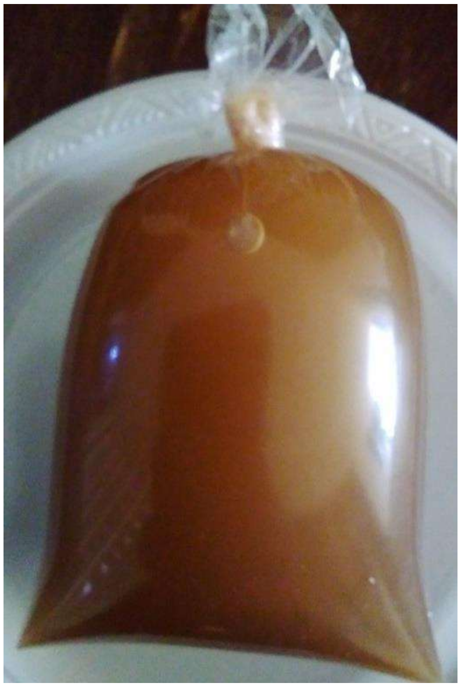

Tamarind drink = 335 mL
= 1 adult serving
= 2.75 child servings

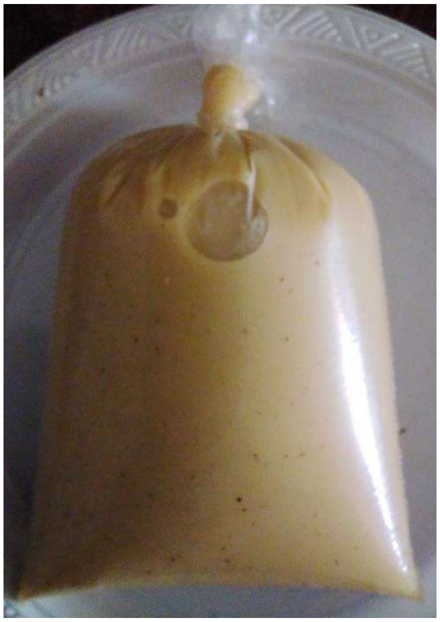

Ice kenkey bag = 275 mL
= 0.75 adult serving
= 2.25 child servings

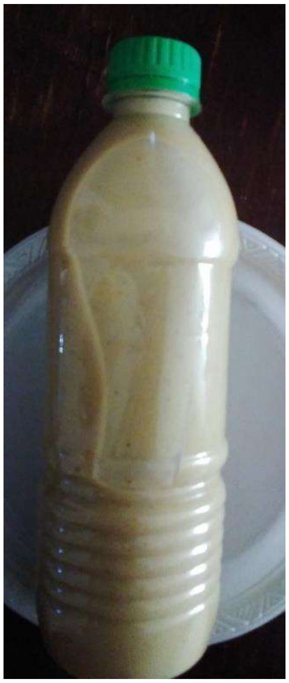

Ice kenkey bottle = 540 mL
= 1.5 adult servings
= 4.5 child servings

Servings of hot beverages sweetened with sugar (for example coffee, tea, hot chocolate or hot milk where sugar or honey was added).

- Adult serving size: 360 mL
- Child serving size: 120 mL

360 mL =

120 mL =

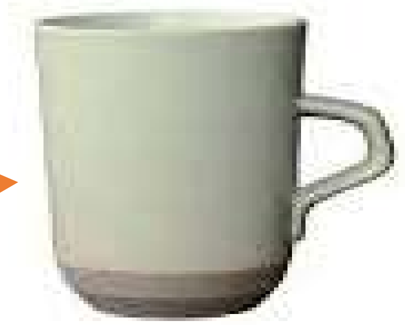

Servings of salty snacks (crisps, popcorn, chips, etc.)

- Adult servings size = 30 g
- Child serving size = 20 g

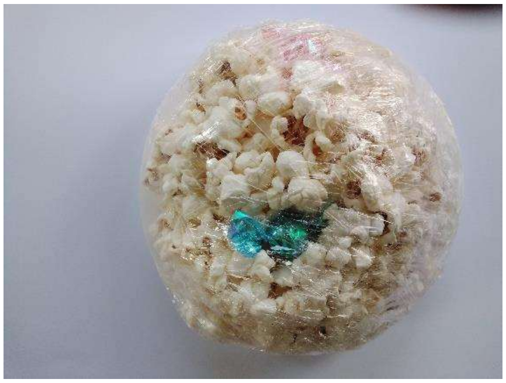

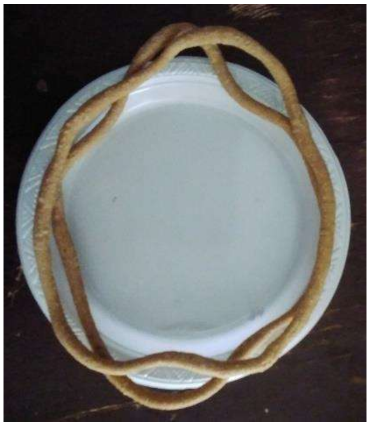

Kulikuli
2 = 34g = 1 adult serving
1 = 17g = 1 child serving

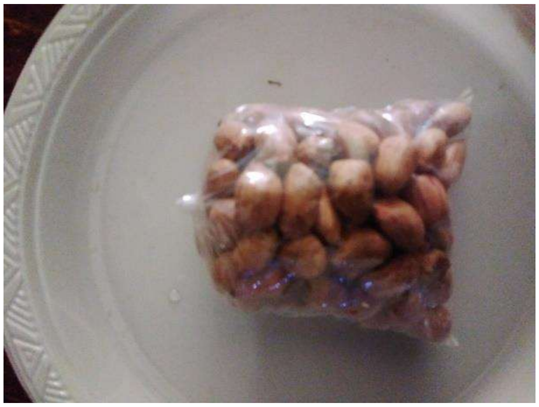

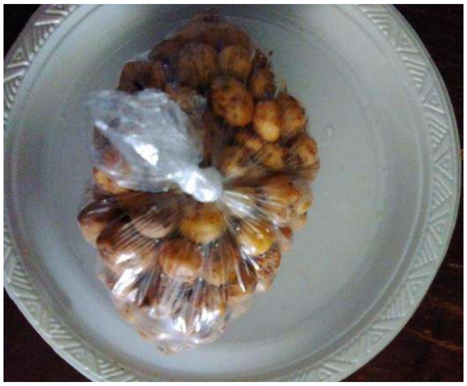

Roasted peanuts = 53 g
= 1.5 adult servings
= 2.5 child servings

Tigernuts = 177 g
= 6 adult servings
= 9 child servings
