# Supplementary material for: Consumption of Selected Healthy and Unhealthy Food Groups and Associations With Nutritional Status Among Children 2–5 Years of Age in Northern Ghana
Source: Matern Child Nutr. 2025 Nov 18;22(1):e70126. doi: 10.1111/mcn.70126 (PMC12624276; doi:10.1111/mcn.70126)
Supplement: Supplementary file 3 — Supplemental‐File‐2_Adapted‐Diet‐Questions1. [file MCN-22-e70126-s003.pdf]

CoMIT Pilot Child Dietary Patterns Manuscript Supplemental File 2
Adapted WHO STEPS Questionnaire Diet Questions

frt1	1. In a typical week, on how many days does your child eat fruit? (USE SHOWCARD)<br><br>**ENTER 99 FOR DON'T KNOW**<br><br>**IF 0 or 99, SKIP TO QUESTION 6.**	|___|___|	
frt2	2. How many **servings** of fruit does your child eat on **one** of those days?<br>(USE SHOWCARD)<br><br>**ENTER 99 FOR DON'T KNOW**	|___|___|	
veg1	3. In a typical week, on how many days does your child eat vegetables?<br>(USE SHOWCARD)<br><br>**ENTER 99 FOR DON'T KNOW**<br><br>**IF 0 or 99, GO TO QUESTION 8.**	|___|___|	
veg2	4. How many **servings** of vegetables does your child eat on **one** of those days?<br>(USE SHOWCARD)<br><br>**ENTER 99 FOR DON'T KNOW**	|___|___|	
sltsnack1	5. In a typical week, on how many days does your child eat salty snacks, like crisps or salty crackers?<br>(USE SHOWCARD)<br><br>**ENTER 99 FOR DON'T KNOW**<br><br>**IF 0 or 99, GO TO QUESTION 10.**	|___|___|	
sltsnack2	6. How many **servings** of salty snacks does your child eat on **one** of those days?<br>(USE SHOWCARD)<br><br>**ENTER 99 FOR DON'T KNOW**	|___|___|	
swtsnack1	7. In a typical week, on how many days does your child sweet snacks, like biscuits, candies or chocolates?<br>(USE SHOWCARD)<br><br>**ENTER 99 FOR DON'T KNOW**<br><br>**IF 0 or 99, GO TO QUESTION 12.**	|___|___|	
swtsnack2	8. How many **servings** of sweet snacks does your child eat on **one** of those days?<br>(USE SHOWCARD)<br><br>**ENTER 99 FOR DON'T KNOW**	|___|___|	

ssb1	9. In a typical week, on how many days your child drink beverages sweetened with sugar, like soda or sugar-sweetened fruit drinks?<br>(USE SHOWCARD)<br><br>**ENTER 99 FOR DON'T KNOW**<br><br>**IF 0 or 99, GO TO QUESTION 14.**	|___|___|	
ssb2	10. How many **servings** of beverages sweetened with sugar does your child drink on **one** of those days?<br>(USE SHOWCARD)<br><br>**ENTER 99 FOR DON'T KNOW**	|___|___|	
ssb3	11. In a typical week, on how many days does your child drink hot beverages sweetened with sugar, like sweetened milk drinks?<br>(USE SHOWCARD)<br><br>**ENTER 99 FOR DON'T KNOW**<br><br>**IF 0 or 99, GO TO QUESTION 16**	|___|___|	
ssb4	12. How many **servings** of hot beverages sweetened with sugar does your child drink on **one** of those days?<br>(USE SHOWCARD)<br>**ENTER 99 FOR DON'T KNOW**	|___|___|	
